# Locally advanced breast cancer made amenable to radical surgery after a combination of systemic therapy and Mohs paste: two case reports

**DOI:** 10.1186/1752-1947-6-360

**Published:** 2012-10-24

**Authors:** Tomoya Tsukada, Tatsuo Nakano, Miki Matoba, Daisuke Matsui, Shozo Sasaki

**Affiliations:** 1 Department of Gastroenterological Surgery, Division of Cancer Medicine, Graduate School of Medical Science, Kanazawa University, 13-1 Takara-machi, Kanazawa, Ishikawa 920-8641, Japan; 2 Department of Surgery, Asanogawa General Hospital, Kanazawa, Ishikawa 920-8621, Japan

**Keywords:** Breast cancer, Modified radical mastectomy, Mohs surgery

## Abstract

**Introduction:**

Chemotherapy and other systemic therapies are the primary treatments for patients with unresectable, locally advanced breast cancer. The clinical application of supportive care using Mohs paste has become widespread for the purpose of improving patients’ quality of life. Here, we report two cases of locally advanced breast cancer, for which the patients underwent radical surgery after a combination of systemic therapy and Mohs chemosurgery.

**Case presentations:**

Patient 1 was a 90-year-old Japanese woman with right breast cancer diagnosed as stage IIIB (T4bN1M0). The treatment included Mohs paste application and hormonal therapies. Patient 2 was a 60-year-old Japanese woman with right breast cancer diagnosed as stage IIIB (T4cN2aM0). Her treatment included Mohs paste application, together with chemotherapy (four cycles of 5-fluorouracil, epirubicin, and cyclophosphamide, and four cycles of docetaxel). In both cases, a reduction in the primary tumor volume was observed, and radical mastectomy and axillary lymph node dissection were possible without relaxation incision or skin flap.

**Conclusion:**

We report patients with no distant metastases who were able to undergo radical resection after a combination of systemic therapy and Mohs chemosurgery. For locally advanced breast cancer, Mohs chemosurgery, in addition to multidisciplinary treatment, is useful.

## Introduction

For patients with unresectable, locally advanced breast cancer with skin invasion, the primary therapy is systemic, such as chemotherapy [1]. However, the presence of bleeding, exudates, and/or strong odor from infection can adversely impact quality of life (QOL). These symptoms are collectively referred to as malignant wounds and are managed with palliative treatment [2,3]. Patients with breast, head and neck, and primary skin cancers have the highest prevalence of malignant wounds (47.1%, 46.7%, and 39.1% of patients, respectively) [4], and Mohs chemosurgery has been applied for such patients [5-7]. Mohs chemosurgery is a technique of chemical fixation of a cutaneous tumor and subsequent excision [8,9]. The fixative used in a Mohs procedure contains zinc chloride and is referred to as Mohs paste. In this technique, fixation and excision are repeated until no residual tumor can be found in the specimen by microscopic examination [10]. In recent years, the use of Mohs chemosurgery has become widespread for the primary purpose of improving QOL. However, in patients with breast cancer, there is little reported experience with the application of Mohs paste for the purpose of tumor reduction. To the best of our knowledge, there are no reports of patients who underwent radical surgery after a combination of systemic therapy and Mohs chemosurgery. Here, we report two cases of locally advanced breast cancer for which the patients underwent radical surgery after a combination of systemic therapy and Mohs chemosurgery. Furthermore, as frequent dressing changes are necessary due to exudates from ulcers after Mohs chemosurgery, we report easy management of these issues in the out-patient setting by an appropriate choice of wound dressing.

After obtaining approval from the Committee on Pharmaceutical Affairs in our hospital and written informed consent from the patients, we obtained Mohs paste formulated by the pharmaceutical department. Zinc chloride was ground into a powder in a mortar and dissolved using purified water. Next, zinc oxide starch powder was mixed gradually. Finally, glycerin was added to a viscosity individualized according to the patient’s need (Table 1). Mohs paste was formulated on the day of use. After petroleum jelly was applied to the surrounding normal skin, Mohs paste was applied to the tumor and covered with gauze. Mohs paste was removed 24 hours after application, and petroleum jelly was reapplied. Gauze was changed every day. Necrotic tissue was removed bluntly. This method was repeated until the tumor flattened (Table 2).

**Table 1:** Formulation of Mohs paste

**Material**	**Original method**^**a**^	**Our hospital**
Saturated zinc chloride	34.5mL	
Zinc chloride		10g
Purified water		10mL
Powdered *Sanguinaria canadensis*	10g	
Zinc oxide starch powder		5g
Paste containing stibnite	40g	
Glycerin		5–10mL

^a^Original method [8,9].

**Table 2:** Protocol for the Mohs method in our hospital

	
1.	Apply petroleum jelly to the surrounding normal skin, and cover with gauze to protect from Mohs paste.
2.	Uniformly apply the Mohs paste at a thickness of 1mm on the surface of tumor and protect using gauze.
3.	Remove the gauze and Mohs paste 24 hours after application.
4.	After removal of Mohs paste, apply petroleum jelly and protect using gauze.
5.	Observe the tumor at the same time every day. Necrotic tissue may be removed bluntly, or it might fall off naturally.
6.	Continue above cycle every 2–3 days until the elevated tumor is flattened.

## Case presentations

### Patient 1

**Figure 1:**
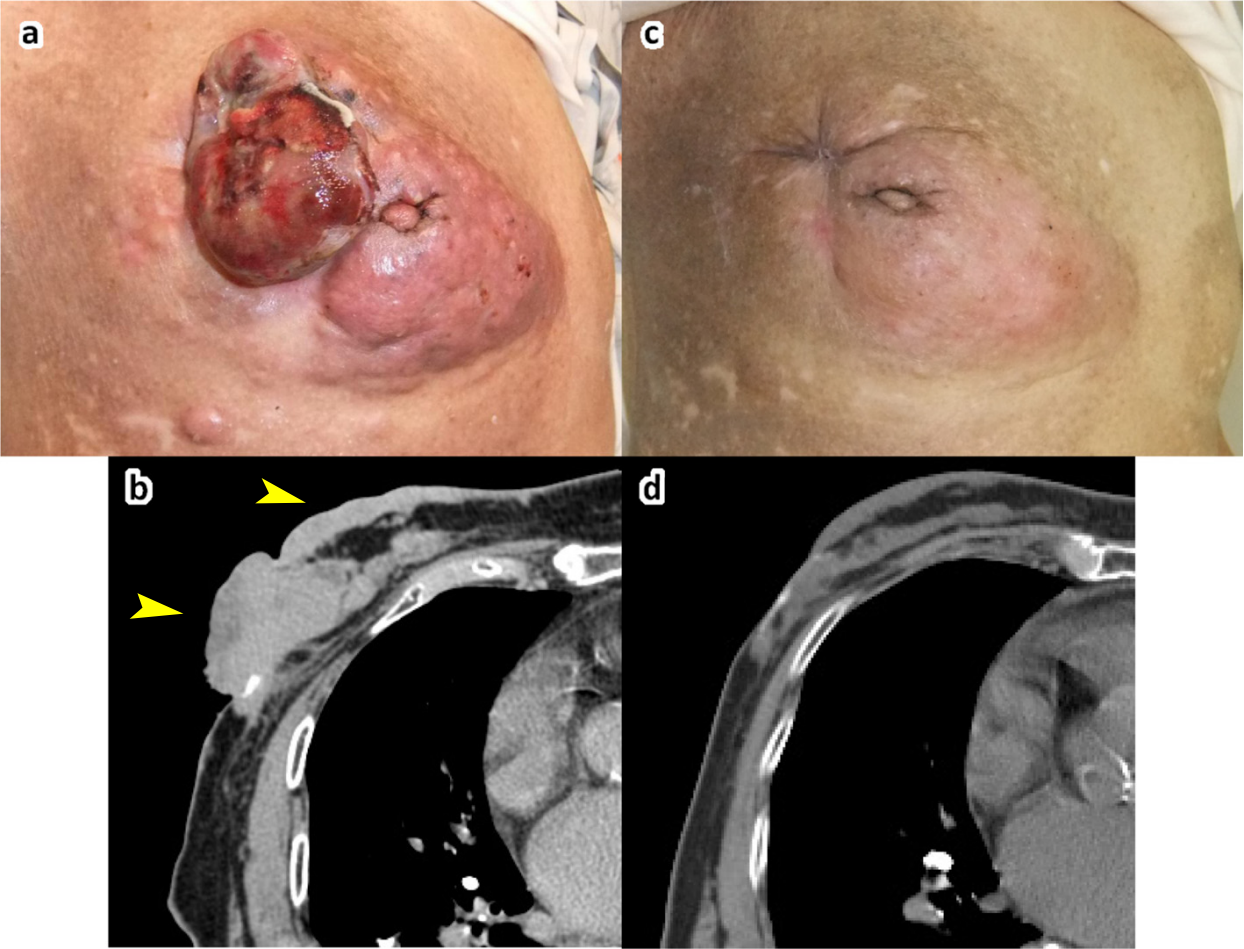
**Case 1.a**: Macroscopic image of the right breast at the initial visit. **b**: Computed tomography (CT) findings at the initial visit. CT revealed a mass with overlying and surrounding skin thickening (arrowheads). **c**: Macroscopic image of the right breast before surgical treatment. **d**: CT findings before surgical treatment.

A 90-year-old Japanese woman presented with back pain, and a right breast mass was found at that time. She had been aware of bleeding from the breast mass for the past 5 years. The mass was an 8cm-diameter tumor of the right breast, measured from the nipple laterally and including the central portion of the breast. There was infiltration into the skin, but no fixation to the chest wall was observed (Figure 1a). She also reported tenderness in the thoracic vertebrae. A computed tomography (CT) scan revealed a huge mass in the right breast with overlying and surrounding skin thickening (Figure 1b). A swollen axillary lymph node and a compression fracture in the twelfth thoracic vertebra were also observed. Magnetic resonance imaging (MRI) was also performed for the vertebra and neoplastic lesions were not obvious. A core needle biopsy was performed, and histopathologic examination of the biopsy specimen revealed invasive micropapillary carcinoma, estrogen receptor (ER)-positive, progesterone receptor (PgR)-positive, and human epidermal growth factor receptor 2 (HER2)-1+. ER, PgR and HER2 status was determined using immunohistochemical examination and/or fluorescence *in situ* hybridization (FISH). ER and PgR results were considered positive with ≥10% positively staining cells. HER2 was considered positive with either 3+ immunoreactivity or amplification by FISH in accordance with the American Society of Clinical Oncology-College of American Pathologists guideline [11]. Positron emission tomography (PET)-CT was performed, and the final diagnosis was breast cancer, stage IIIB (T4bN1M0).

The patient was hospitalized, with bedrest as treatment for the compression fracture, and letrozole was selected as systemic therapy for the breast cancer. Alendronate, calcium L-aspartate and alfacalcidol were also administered. Written informed consent was obtained from the patient, and Mohs paste was applied to control local bleeding and exudates. After using Mohs paste for three cycles, the tumor flattened, and there was no further need for daily dressing changes. Versiva® XC® (ConvaTec Japan, Tokyo, Japan), which contains hydrocolloid and hydrofiber, was used for wound closure [12]. The Versiva® XC® dressing required once-weekly dressing changes. The wound could be left uncovered starting on day 28 from the first Mohs chemosurgery. Letrozole was continued for approximately 6 months in the out-patient setting. The skin infiltration reduced gradually (Figures 1c, 1d). Radical mastectomy of the right breast and axillary lymph node dissection (level 1) were performed. The infiltrated skin was resected at the same time, without the need for relaxation incision or skin flap. Histologic examination showed mucinous carcinoma with lymph node metastasis (metastatic axillary lymph nodes/dissected lymph nodes: 3/26); the histologic evaluation of the therapeutic effect was grade 1b. The patient was discharged on postoperative day 16 without any postoperative complications. She has continued oral letrozole since her surgery of 15 months ago.

### Patient 2

**Figure 2:**
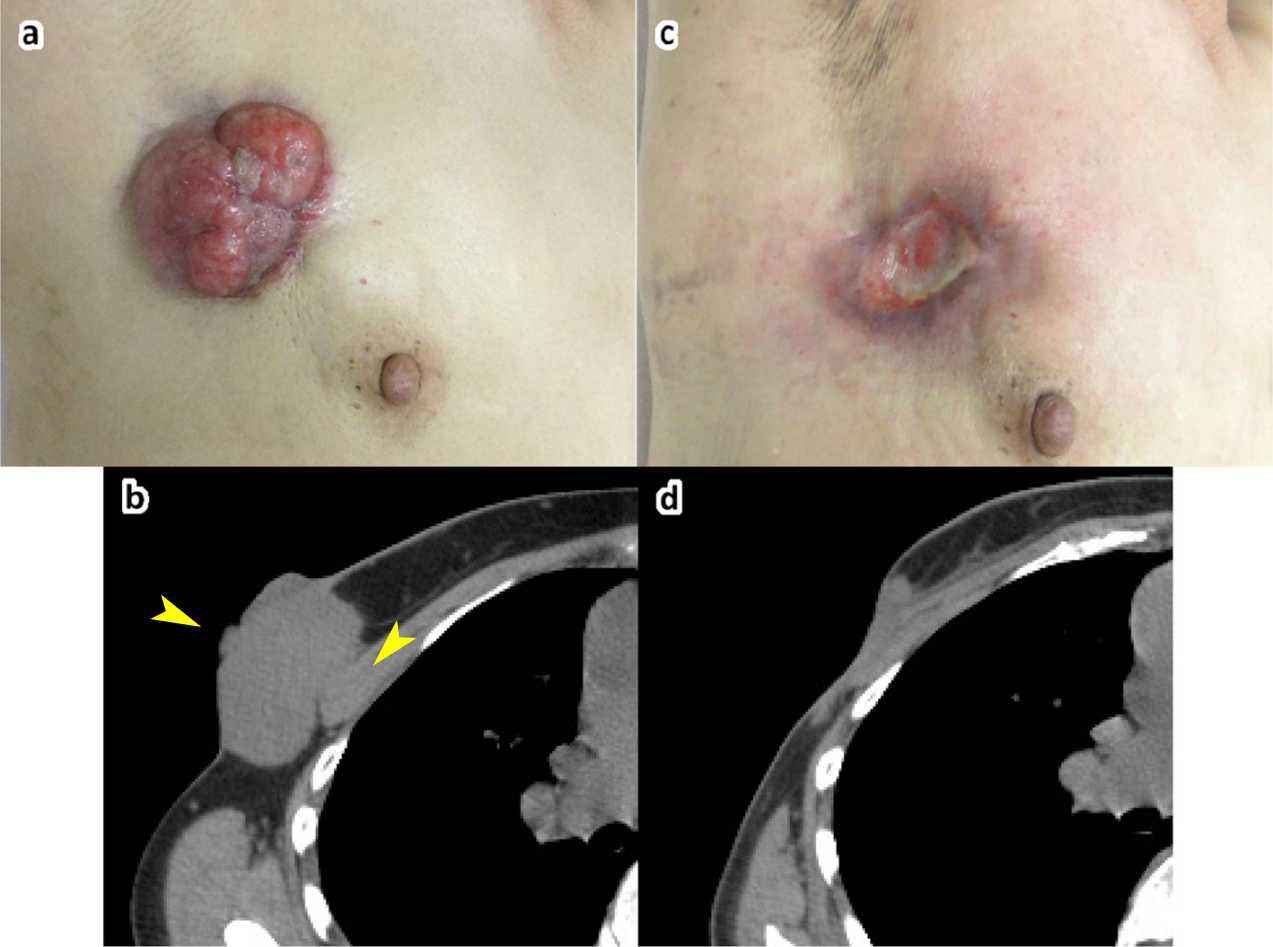
**Case 2.a**: Macroscopic image of the right breast at the initial visit. **b**: Computed tomography (CT) findings at the initial visit. CT revealed a mass with infiltration of the pectoralis major muscle (arrowheads). **c**: Macroscopic image of the right breast before surgical treatment. **d**: CT findings before surgical treatment.

A 60-year-old Japanese woman presented with a right breast mass with exudates. She had been aware of the breast mass for the past 5 years. A 5cm-diameter tumor was present in the superior-lateral quadrant of the right breast. Exposure to the skin and fixation to the chest wall were observed (Figure 2a). Hard axillary lymph nodes were also palpable. CT revealed a huge mass in the right breast with infiltration of the pectoralis major muscle (Figure 2b). Swollen axillary lymph nodes were also observed. A core needle biopsy was performed, and histopathologic examination of the biopsy specimen revealed invasive papillotubular carcinoma, ER-positive, PgR-positive, and HER2-negative. PET-CT was performed, and the final diagnosis was breast cancer, stage IIIB (T4cN2aM0). In addition, idiopathic thrombocytopenia and chronic hepatitis C were diagnosed at this visit.

She underwent four cycles of 5-fluorouracil 500mg/m^2^, epirubicin 100mg/m^2^, and cyclophosphamide 500mg/m^2^ (FEC100) and four cycles of docetaxel (75mg/m^2^) as neoadjuvant chemotherapy. Chemotherapy was initiated with an 80% dose reduction due to pancytopenia from idiopathic thrombocytopenia and chronic hepatitis C. Thereafter she was able to complete therapy without cessation or further reduction of chemotherapy drugs. Written informed consent was obtained from the patient, and Mohs paste was applied for exudates. After using Mohs paste for three cycles, the wound was closed with Versiva® XC® on day 15 from the first Mohs chemosurgery. Although she could have been discharged from the hospital before her the third course of FEC100, weekly dressing changes of the Versiva® XC® were required. After neoadjuvant therapy was changed to docetaxel, tumor reduction occurred slowly, and the tumor did not epithelialize (Figure 2c). After completion of chemotherapy, invasion of the pectoralis major muscle was still present (Figure 2d). Radical mastectomy of the right breast and axillary lymph node dissection (level 2) were performed. The invaded portions of the pectoralis major and pectoralis minor muscles were also resected. Relaxation incision or skin flap was not required. Histologic examination showed papillotubular carcinoma with lymph node metastasis (metastatic axillary lymph nodes/dissected lymph nodes: 4/17), and the histologic evaluation of therapeutic effect was grade 1b. The patient was discharged on postoperative day 24 without any postoperative complications. She began oral letrozole 9 months ago after her surgery.

## Discussion

Mohs chemosurgery is minimally invasive and relatively simple. This method has been applied widely in areas such as palliative care and is considered a very useful treatment [7]. Although no guiding philosophy exists regarding the use interval and contact time with Mohs paste, the progress and depth of consolidation depend on contact time. Reported contact times range from a few minutes to 48 hours [10]. Hemostatic effects have been seen with times ranging from a few minutes to 10 minutes [7]. The intended purpose of Mohs paste application is to determine the appropriate use interval and contact time. When using Mohs paste, it is important to protect normal, healthy skin. Several methods for containing the oil, such as thick application of petroleum jelly or affixing dressing agents, have been applied [7]. For the formulation of Mohs paste, a saturated solution of zinc chloride (purified water:zinc chloride, 1mL:2g) was made, then mixed with zinc oxide starch powder, and the viscosity was adjusted using glycerin. This preparation method allowed the production of a roughly common product. With the aim of tumor reduction, a relatively long contact time (24 hours) was determined and the concentration was reduced to 50%, instead of a saturated solution, for the purpose of achieving slow infiltration. Tissue fixation can be obtained even at concentrations of 50%; therefore, a saturated solution was deemed unnecessary. After using Mohs paste, necrotic tissue is formed. In many cases, necrotic tissue is removed bluntly, without local anesthesia, or it falls off naturally. After removal, an ulcer might form, and exudates might reduce; however, regular dressing changes remain necessary.

Closure therapy was performed according to pressure ulcer protocol using Versiva® XC®. Versiva® XC® is an adhesive patch, with reported effectiveness; it creates a moist environment to promote wound healing and can protect the surrounding environment by containing the exudate [13]. Daniels *et al*. demonstrated ease-of-use and no problems with use of the patch for an average of between 5 and 6 days [14]. Similarly, the present patients experienced no problems with weekly dressing changes in the out-patient setting. Complete epithelialization was achieved in Case 1. In Case 2, complete closure was not achieved due to a decline in the antitumor effects of docetaxel, but weekly dressing changes could be continued without complications such as infection. Versiva® XC® is a useful dressing in the out-patient setting.

In breast cancer treatment, few experiences have been reported with the primary goal of cytoreduction. To the best of our knowledge, radical surgery after a combination of systemic therapy and Mohs chemosurgery has not been reported. In skin tumor resection, Mohs chemosurgery is not a first-line choice, but good results have also been reported [5,15,16]. For patients with locally advanced breast cancer, multidisciplinary treatment should be applied. However, Mohs chemosurgery can aid in tumor reduction when combined with multidisciplinary treatment in patients with no distant metastases. In particular, Mohs chemosurgery is a useful method in patients with bleeding and exudates.

## Conclusions

This report demonstrates that for patients with no distant metastases, radical resection through a combination of systemic therapy and Mohs chemosurgery is useful. Mohs paste resulted in tumor reduction, and wound closure was completed under out-patient care. Combination therapy allowed a simple suture closure of the dermis.

For locally advanced breast cancer, Mohs chemosurgery, in addition to multidisciplinary treatment, is a useful approach.

## Consent

Written informed consents were obtained from the patients for publication of these two case reports and accompanying images. Copies of the written consents are available for review by the Editor-in-Chief of this journal.

## Competing interests

The authors declare that they have no competing interests.

## Authors' contributions

TT participated in treatment of the patients, collected case details, conducted the literature search and helped to draft the manuscript. TN participated in treatment of the patients and helped to draft the manuscript. MM, DM and SS participated in treatment of the patients. All authors read and approved the final manuscript.
